# 

**Published:** 1899-11

**Authors:** 


Vol. LIII. NOVEMBER, 1899. No. 11.

LIBRARY
Indexed
[illegible]

### THE DENTAL REGISTER

A MONTHLY JOURNAL.

Devoted to the Interests of the Profession.

EDITED BY

J. TAFT, D.D.S.

LIBRARY

SURGEON GENERAL'S OFFICE

NOV.-24.-1899

PUBLISHED BY
SAM'L A. CROCKER & CO.,
OHIO DENTAL AND SURGICAL DEPOT,
35, 37 and 39 West Fifth Street, CINCINNATI, OHIO.

Entered at the Postoffice at Cincinnati, O., as second class mail matter.

TERMS: $2.00 PER ANNUM IN ADVANCE. SINGLE COPIES, 25 CTS.

